# Rim sign and histogram analysis of apparent diffusion coefficient values on diffusion-weighted MRI in triple-negative breast cancer: Comparison with ER-positive subtype

**DOI:** 10.1371/journal.pone.0177903

**Published:** 2017-05-18

**Authors:** Yangsean Choi, Sung Hun Kim, In Kyung Youn, Bong Joo Kang, Woo-chan Park, Ahwon Lee

**Affiliations:** 1 Department of Radiology, Seoul St. Mary’s Hospital, College of Medicine, The Catholic University of Korea, Seoul, Republic of Korea; 2 Department of General Surgery, Seoul St. Mary’s Hospital, College of Medicine, The Catholic University of Korea, Seoul, Republic of Korea; 3 Department of Hospital Pathology, Seoul St. Mary’s Hospital, College of Medicine, The Catholic University of Korea, Seoul, Republic of Korea; University of Chicago, UNITED STATES

## Abstract

**Purpose:**

To investigate associations between the clinicopathologic features and MRI features of triple-negative breast cancer (TNBC) and ER-positive breast cancer (BC) via apparent diffusion coefficient (ADC) histogram analysis.

**Materials and methods:**

In this study, 221 breast cancer patients with pre-operative MRI performed from August 2009 to March 2015 were included in a retrospective analysis. All patients had a pathologically confirmed diagnosis of invasive carcinoma and were grouped into ER-positive (149) or triple-negative (72) subtypes. DWI rim sign and various ADC parameters (mean; mode; 25, 50, and 75 percentiles; skewness; and kurtosis) between ER-positive and TNBC were compared using whole-lesion ADC histogram analysis. Univariate and multivariate regression analyses were used for statistical comparison.

**Results:**

DWI rim signs were detected in 42.3% and 41.7% of ER-positive subtype and TNBC, respectively (*P* = 0.931). TNBC had poorer histologic grade (*P*<0.001) and higher Ki-67 expression (*P* <0.001) than ER-positive subtype BC. TNBC displayed higher ADC parameters (mean, mode, 50th & 75th percentiles, kurtosis on univariate analysis, all *P*<0.001; only kurtosis on multivariate anaylsis; *P*<0.001) than ER-positive subtype BC. TNBC had significantly more recurrence events than ER-positive subtype BC on univarate analysis (9.7% (7/72) vs. 2.7% (4/149), *P* = 0.035).

**Conclusion:**

Poorer clinicopathologic outcomes were found in TNBC. Whole-lesion ADC histogram analysis revealed ADC kurtosis to be higher in TNBC than ER-positive subtype BC.

## Introduction

Currently, breast cancer is recognized as a group of highly heterogeneous diseases and is further categorized into three major different subtypes based on immunohistochemical expression of receptors: triple-negative [estrogen receptor (ER) negative, progesterone receptor (PR) negative, and human epidermal growth factor receptor 2 (HER2) negative], HER2-positive (HER2+; ER and PR + or -), and ER-positive (ER+, HER2-, PR + or -) [1,2]. Because of this mixed spectrum of gene expression, each subtype displays different clinical behaviors, responses to treatment, and prognosis. [3] In particular, triple-negative breast cancer (TNBC) lacks expression of all three receptors (ER, PR, and HER2) and is known to have a more aggressive clinical course and poorer outcomes. [4–6] Accordingly, early distinction of TNBC from other subtypes with a non-invasive imaging modality using MRI would allow clinicians to establish ideal treatment management before final pathologic confirmation. [7]

Previous studies have described MRI features of TNBC as a larger size and higher apparent diffusion coefficient (ADC) on diffusion-weighted image (DWI) due to a greater necrotic component. [8,9] However, these studies only measured ADC values from a single slice of ADC maps, which could have resulted in observer bias and insufficient information regarding radiologic heterogeneity of the tumor. In order to overcome such limitations, we adopted a volumetric analysis of the entire tumor by mapping ADC histograms. A similar study by Suo et al. [10] demonstrated that whole-lesion ADC histogram analysis could facilitate differentiation between benign and malignant breast mass lesions. Kim et al. found that various ADC histogram parameters correlated with prognostic factors and subtypes of invasive ductal carcinoma (IDC). [11]

This study aimed to investigate associations between TNBC and ER-positive BC with regard to clinicopathologic parameters and MRI features of DWI rim sign and ADC histogram analysis.

## Materials and methods

### Patient selection

The Institutional Review Board of Seoul St. Mary’s Hospital reviewed and approved this retrospective study, and the requirement for informed patient consent was waived. All patients’ data were extracted via electronic charts of our institution and one radiologist (YC) could identify individual patients throughout data collection. A total of 470 breast cancer patients with pathologically proven invasive carcinoma were included. All patients with pre-operative breast MRI performed at 3.0T from August 2009 to March 2015 were retrospectively reviewed through medical records and a PACS (picture archiving and communication system). Of the total patients 31 were excluded due to insufficient information on molecular markers or positive expression of HER2-receptor. Among the remaining 439 patients, 218 were additionally excluded during image analysis due to neoadjuvant chemotherapy (n = 33), image artifact or poor image quality (n = 14), processing software error (n = 21), small tumor size (<1cm) (n = 129), and non-mass enhancement (n = 21), leaving 221 invasive carcinoma patients consisting of 149 ER-positive and 72 TNBC subtypes for analysis (mean age, 52.3 years, age range, 31–76 years) (Table 1).

**Table 1:** Clinicopathologic characteristics of all patients.

Variable	N (%) or mean±SD
Total	221
Clinicopathologic Characteristics	
Age	
mean±SD	52.28 ± 9.54
median (IQR)	52 (45, 59)
Subtype	
ER-Positive	149 (67.42)
Triple-Negative	72 (32.59)
Histologic Grade	
well, moderate	135 (61.09)
poor	86 (38.91)
Axillary Nodal Status	
negative	142 (64.25)
positive	79 (35.75)
Ki67	
mean±SD	32.11 ± 30.34
median (IQR)	15 (7, 60)
<14%	109 (49.32)
≥14%	112 (50.68)
Lesion size	
mean±SD ER-Positive Triple-Negative	2.29 ± 1.15 2.36 ± 1.23 2.13 ± 0.92
median (IQR)	2.1 (1.5, 2.6)
≤2cm	108 (48.87)
>2cm	113 (51.13)
Recurrence	
none	210 (95.02)
event	11 (4.98)
Follow-up time in months mean±SD	32.95±13.55
median (range)	32 (5, 55)

Values are number (percentage) for categorical variables and mean (SD),or median (IQR) for others.

ER: estrogen receptor; DWI: diffusion-weighted image; ADC: apparent diffusion coefficient

### MRI protocol

Each MRI was performed with the patient in a prone position using a dedicated bilateral breast surface coil. Images were obtained with a 3 T MRI system (Verio; Siemens Healthcare, Erlangen, Germany) using the following sequences: 1) axial, turbo spin-echo T2WI sequence with a TR/TE of 4530/93, flip angle of 80°, 34 slices, FOV of 320 mm, matrix size of 576 × 403, 1 NEX, slice thickness of 4 mm, and acquisition time of 2 minutes, 28 seconds; 2) axial DWI with readout segment echo planar imaging (rs EPI) (b values of 0 and 750 s/mm^2^, TR/TE 5600/55 ms, FOV 360 × 180 mm, matrix size 192 × 82, slice thickness of 4 mm, acquisition time of 2 minutes and 31 seconds, with 5 readout segments); 3) pre- and postcontrast axial T1-weighted flash 3-dimensional VIBE sequences with a TR/TE of 4.4/1.7, flip angle of 10°, slice thickness of 1.2 mm, and acquisition time of 1 minute. The images were obtained before and 70, 130, 190, 250, and 310 seconds after injection of Gd-DTPA (0.1 mmol/kg Gadovist; Bayer Schering Pharma, Berlin, Germany). ADC maps were automatically calculated from the DW images using MRI software.

### Imaging analysis

**Fig 1:**
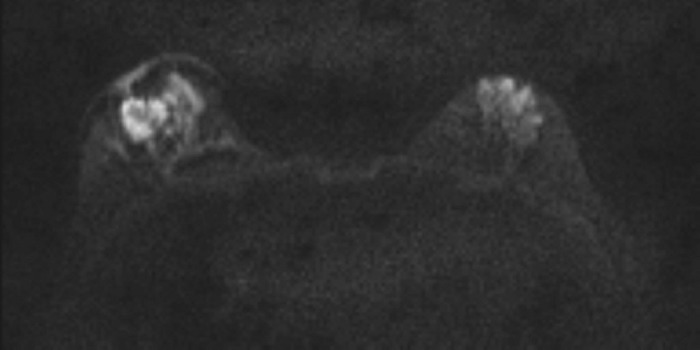
Representative case of positive DWI rim sign. Diffusion weighted image (b = 750 s/mm^2^) shows a complete high signal rim surrounding the right breast mass.

MRI data were evaluated in consensus by two radiologists with 11 years and one year of experience with breast MRIs (SHK, IKY), who were blinded to clinical information of intrinsic subtype and recurrence. The presence of rim sign on DWI was assessed based on the previous definition by Kang et. al [12], who described it as a high signal rim on DWI outlining ≥90% (complete) or ≤90% (incomplete) of the lesion(Fig 1). The absence of rim sign was defined as no visible high signal rim on DWI outlining the lesion.

**Fig 2:**
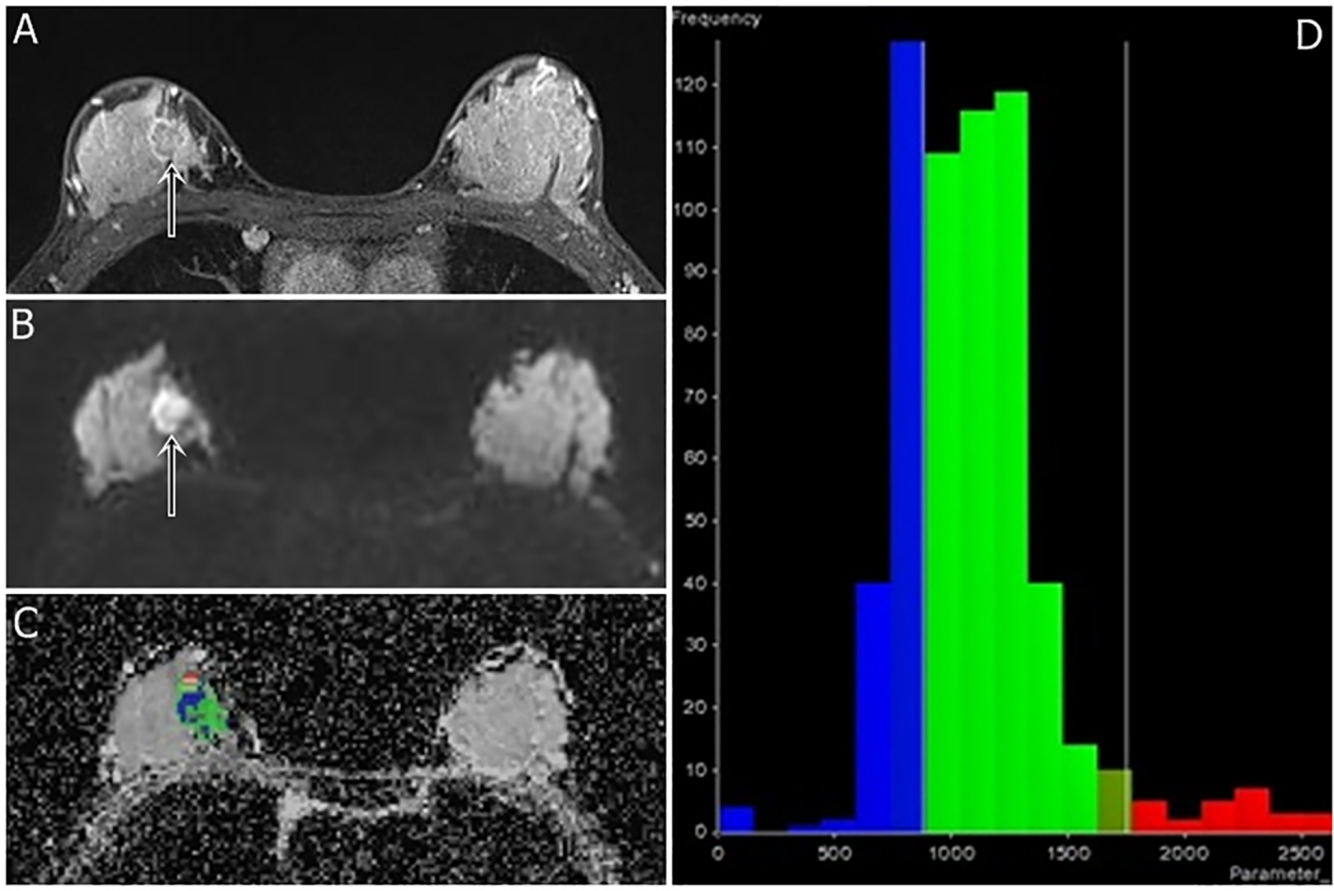
A 41-year-old woman with triple-negative cancer in the right breast (poorly differentiated grade, high Ki-67 index, and high ADC kurtosis). Fat-suppressed contrast-enhanced T1-weighted imaging (a) shows an irregular rim-enhancing mass (arrow). A DWI (b = 750 s/mm^2^) (b) shows positive rim sign (arrow). DWI slice of tumor volume reconstruction of ADC values (c) and a histogram map (d) are shown. ADC mean; mode; and 25^th^, 50^th^, and 75^th^ percentiles were 1.099, 0.745, 0.859, 1.075, and 1.245 x 10^−3^ mm^2^/s, respectively. The ADC skewness and kurtosis were 1.59 and 4.28, respectively. The 3-cm-sized tumor had a poor histologic grade and high Ki-67 index (50%).

**Fig 3:**
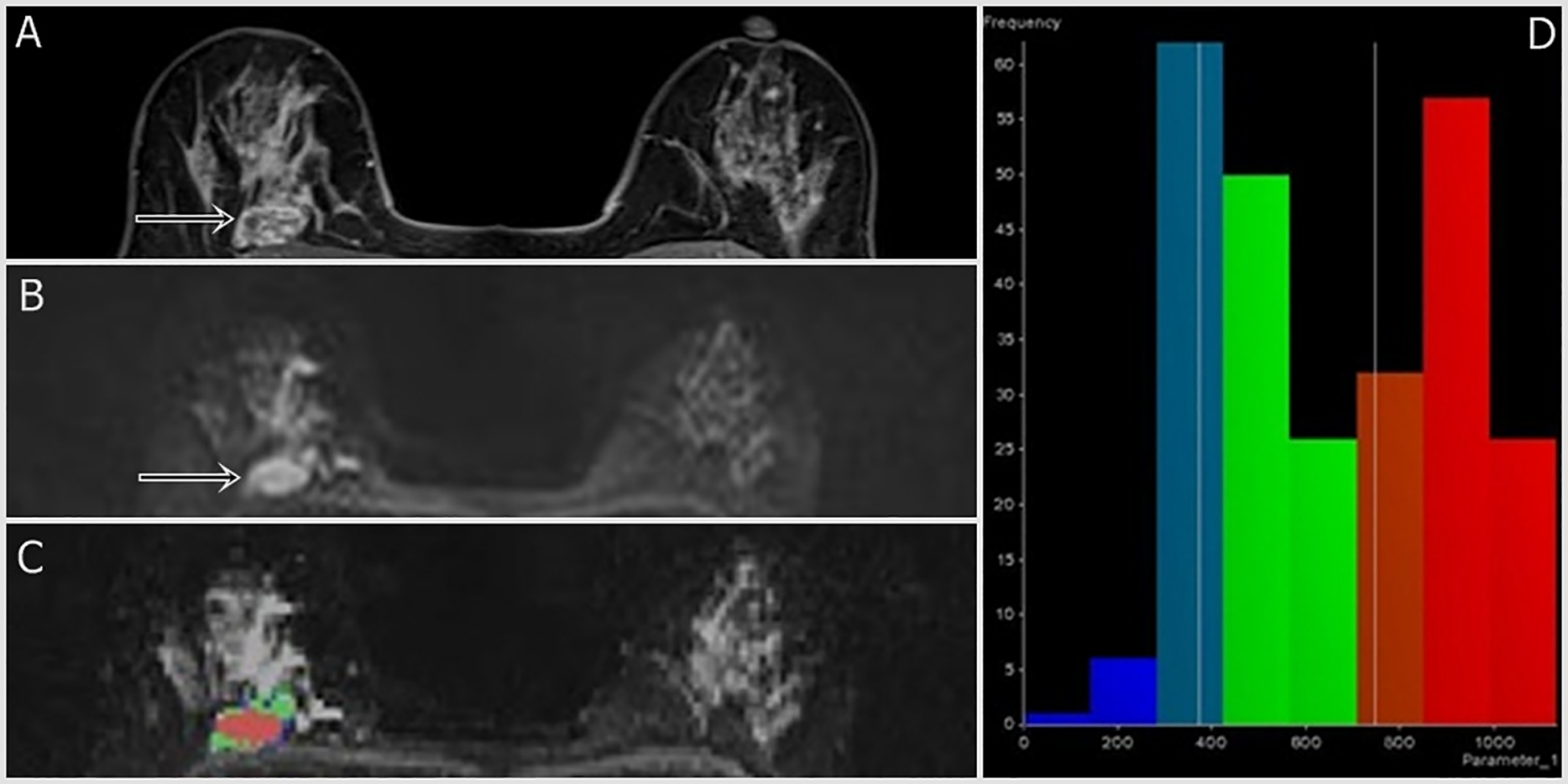
A 45-year-old woman with ER-positive cancer in the right breast (well differentiated grade, low Ki-67 index, and low ADC kurtosis). Fat-suppressed contrast-enhanced T1-weighted imaging (a) shows an irregular heterogeneously enhancing mass (arrowhead). An ADC map (b) shows positive rim sign (arrow). DWI with tumor volume reconstruction of ADC values (c) and a histogram map (d) are shown. ADC mean; mode; and 25^th^, 50^th^, and 75^th^ percentiles were 0.653, 0.389, 0.414, 0.603, and 0.908 x 10^−3^ mm^2^/s, respectively. The ADC skewness and kurtosis were 0.14 and -1.39, respectively. The 2-cm-sized tumor had a well-moderate histologic grade and low Ki-67 index (10%).

We adopted the image analysis method of Kim et. Al. [11] On MRI, dynamic contrast-enhanced images were reviewed as references for tumor detection. In multifocal or multicentric breast cancers, only the largest lesion was selected. The whole lesion was analyzed regardless of cystic, necrotic, or hemorrhagic components in order to evaluate heterogeneity. MR OncoTreat software (Siemens Healthcare, Erlangen, Germany) was used for image analysis. The region of interest was manually drawn by including the index tumor and its margin on DWI (b value = 750 s/mm^2^) sequences from representative axial, sagittal, and coronal images. Difficulty in manual selection of small breast tumors (< 1cm) and non-mass enhancement lesions led to their exclusion in patient selection. Reconstruction of the entire tumor volume, the ADC value of each voxel, and various ADC histogram parameters (mean; 25^th^, 50^th^, and 75^th^ percentiles; mode; skewness; and kurtosis) were generated by OncoTreat. Representative reconstructed images of TNBC and ER-positive BC are shown in Figs 2 and 3, respectively.

### Clinicopathologic data analysis

The radiologist who didn’t participate in image analysis (YC) collected clinical and histopathologic data by reviewing medical records and pathological reports, including patient age, tumor type, size, histological grade, Ki-67 index, presence of axillary lymph node metastasis and presence of recurrence. Tumor size was measured as the maximum diameter of the surgically resected specimen.

On the basis of receptor expression status, tumors were grouped as triple-negative or ER-positive subtype. Histopathological assessment was completed by a pathologist with 15 years of experience. ER and progesterone positivity were defined as more than 1% staining of nuclei in cancer cells on an entire stained slide. The intensity of HER2 expression was semi-quantitatively scored as 0, 1+, 2+, or 3+ such that 3+ score was classified as HER2 positive, and 0 or 1+ score was considered HER2 negative. Gene amplification via dual-color silver in situ hybridization (SISH) with an automated Ventana INFORM HER2 Genomic probe platform (Tucson, Arizona, USA) was performed to determine HER2 status in cancers with a score of 2+.

We defined recurrence as post-operative occurrence of locoregional recurrence, contralateral breast cancer, or distant metastasis. Ipsilateral breast recurrence and regional recurrences in the axilla, chest wall, internal mammary lymph nodes, or supraclavicular lymph nodes were counted as locoregional recurrence. The reported date of pathological diagnosis was considered as the date of recurrence. A few recurrences such as brain metastasis were difficult to be pathologically confirmed and were instead diagnosed by combining multiple imaging modalities such as positron emission tomography-computed tomography (PET-CT) and MRI. In such cases, the reported date of diagnosis by imaging modalities was counted as the date of recurrence. To assess the recurrence-free survival period, we reviewed the medical records to determine the most recent visit date of each patient without recurred disease at our institution (Table 1).

### Statistical analysis

A total of 221 triple-negative or ER-positive breast cancer patients with pre-operative MRI were dichotomized into two groups according to histologic grade (well or moderate vs. poor), axillary nodal status (negative vs. positive), lesion size (≤2cm vs. >2cm), Ki-67 index (<14% vs. ≥14%), and recurrence (event vs. non-event) (Table 1). Clinicopathologic characteristics and MRI features were denoted as n (%) or mean±SD. Associations between the two subtypes for clinicopathologic characteristics and MRI features—DWI rim sign and ADC values—were evaluated by both univariate and multivariate logistic regression. In multivariate logistic regression model, variables that were significant with Bonferroni *post hoc* correction from univariate logistic regression model were included. Statistical analysis was performed with commercially available software (R, v. 3.3.1; R Foundation for Statistical Computing, Vienna, Austria). Differences were considered to be statistically significant at *p*<0.05.

## Results

### Patient characteristics

A total of 221 patients with a mean age of 52.3 years (range, 31–76 years) were included. There were 149 (67.42%) ER-positive and 72 (32.59%) TNBC patients. Well or moderate histologic grade was found in 135 (61.09%) patients, and poor histologic grade was found in 86 (38.91%). In addition, 142 (64.25%) patients had negative axillary nodal status, and 79 (35.75%) patients had positive axillary nodal status. Of the total, 109 (49.32%) patients displayed higher than 14% Ki-67 expression, while 112 (50.68%) displayed less than 14% Ki-67 expression (mean Ki67, 32.11±30.34; median, 15; IQR, 7–60). The histologic size of tumors ranged from 1–9 cm (mean tumor size, 2.29±1.15 cm; median, 2.1 cm). The mean sizes of TNBC and ER-positive subtype were 2.13±0.92 cm and 2.36±1.23 cm, respectively. There were 11 (4.98%) recurrent events, three of which were distant metastasis to bones, two to the lungs, three with local breast tumor recurrence, and one each was metastasis in the chest wall, a cervical lymph node, or an axillary lymph node. The follow-up period for all patients ranged from 5–55 months (mean, 32.95±13.55 months; median, 32 months).

### Associations of clinicopathologic features between the two subtypes

Table 2 shows the results of univariate and multivariate analyses comparing the clinicopathologic features associated with triple-negative and ER-positive subtypes. Histologic grade and Ki-67 value were significant factors in both univariate and multivariate analyses. Poorer histologic grade of tumor was significantly associated with TNBC subtype (15.4% (23/149) in ER-positive vs. 87.5% (63/72) in TN, p<0.001). Higher Ki-67 was significantly associated with TNBC subtype (Ki-67≥14% in 30.2% (45/149) of ER-positive vs. 93.1% (67/72) of TN, *p*<0.001). The TN subtype had significantly more recurrence events than the ER-positive subtype (9.7% (7/72) vs. 2.7% (4/149), p = 0.035), but the difference did not reach statistical significance in multivariate analysis (*p* = 0.293). No significant difference was found in axillary nodal status or lesion size between the two subtypes.

**Table 2:** Clinicopathologic associations between triple-negative and ER-positive breast cancers.

			Univariate		Multivariate	
Variable	ER-Positive (n = 149)	Triple-Negative (n = 72)	OR (95% CI)	*p* value	OR (95% CI)	*p* value
Age			1.00 (0.97, 1.03)	0.765		
mean±SD	52.41 ± 9.26	52 ± 10.17				
median (IQR)	52 (46, 60)	52 (45, 58.25)				
Histologic Grade						
well, moderate	126 (84.56)	9 (12.5)	Reference			
poor	23 (15.44)	63 (87.5)	38.35 (17.52, 92.89)	<0.001	12.96 (5.20, 35.32)	**<0.001**
Axillary Nodal Status						
negative	94 (63.09)	48 (66.67)	Reference			
positive	55 (36.91)	24 (33.33)	0.85 (0.47, 1.54)	0.603		
Ki67						
mean±SD	16.47 ± 17.64	64.47 ± 25.08				
median (IQR)	10 (5, 25)	70 (48.75, 80)				
<14%	104 (69.80)	5 (6.94)	Reference			
≥14%	45 (30.20)	67 (93.06)	30.97 (12.77, 92.97)	<0.001	7.16 (2.30, 25.20)	**<0.001**
Lesion Size						
mean±SD	2.36 ± 1.24	2.13 ± 0.92				
median (IQR)	2.1 (1.5, 2.7)	1.95 (1.5, 2.5)				
≤2cm	68 (45.64)	40 (55.56)	Reference			
>2cm	81 (54.36)	32 (44.44)	0.67 (0.38, 1.18)	0.168		
Recurrence						
none	145 (97.32)	65 (90.28)	Reference			
event	4 (2.68)	7 (9.72)	3.9 (1.14, 15.34)	**0.035**	2.78 (0.49, 21.95)	0.293

OR: odds ratio

### Associations of MRI features between the two subtypes

There was no significant difference in DWI rim sign between the two subtypes (42.28% (63/149) in ER-positive and 41.67% (30/72) in TNBC, p = 0.931) (Table 3). On univariate analysis, higher ADC values were significantly associated with TNBC subtype (mean, mode, 25, 50 percentile, kurtosis; all p<0.001), but no significant association was found for the ADC 75th percentile or skewness (Table 3). On multivariate analysis, only ADC kurtosis was significantly higher in TNBC than the ER-positive subtype (p<0.001).

**Table 3:** Associations of MRI features between triple-negative and ER-positive breast cancers.

			Univariate		Multivariate	
Variable	ER-Positive (n = 149)	Triple-Negative (n = 72)	OR (95% CI)	*p* value	OR (95% CI)	*p* value
DWI rim						
negative	86 (57.72)	42 (58.33)	Reference			
positive	63 (42.28)	30 (41.67)	0.98 (0.55, 1.72)	0.931^**a**^		
ADC mean (10^-3^mm^2^/s)			1.002 (1.001, 1.003)	**<0.001**^**a**^^,^^**b**^	1.0008 (0.99, 1.007)	0.816^**a**^
mean±SD	0.962 ± 0.366	1.129 ± 0.181				
median (IQR)	1.054 (0.678, 1.187)	1.119 (0.997, 1.231)				
ADC mode (10^-3^mm^2^/s)			1.0013 (1.0005, 1.0022)	**0.002**^**a**^^,^^**b**^	0.9997 (0.998, 1.001)	0.686^**a**^
mean±SD	0.813 ± 0.386	0.976 ± 0.286				
median (IQR)	0.863 (0.547, 1.050)	0.979 (0.800, 1.150)				
ADC 25 percentile (10^-3^mm^2^/s)			1.002 (1.001, 1.004)	**<0.001**^**a**^^,^^**b**^	0.999 (0.995, 1.003)	0.814^**a**^
mean±SD	0.753 ± 0.306	0.914 ± 0.187				
median (IQR)	0.823 (0.532, 0.957)	0.926 (0.767, 1.014)				
ADC 50 percentile (10^-3^mm^2^/s)			1.002 (1.001, 1.003)	**<0.001**^**a**^^,^^**b**^	1.002 (0.994, 1.01)	0.616^**a**^
mean±SD	0.937 ± 0.361	1.101 ± 0.190				
median (IQR)	1.032 (0.676, 1.162)	1.081 (0.981, 1.198)				
ADC 75 percentile (10^-3^mm^2^/s)			1.0011 (1.0003, 1.0019)	0.007^**a**^		
mean±SD	1.156 ± 0.448	1.310 ± 0.212				
median (IQR)	1.246 (0.827, 1.440)	1.293 (1.179, 1.438)				
ADC skewness			1.86 (1.18, 3)	0.008^**a**^		
mean±SD	0.29 ± 0.57	0.53 ± 0.73				
median (IQR)	0.25 (-0.03, 0.64)	0.48 (0.11, 0.97)				
ADC kurtosis			1.67 (1.38, 2.06)	**<0.001**^**a**^^**,**^^**b**^	1.71 (1.37, 2.19)	**<0.001**^**a**^^**,**^^**b**^
mean±SD	0.39 ± 1.46	1.79 ± 1.92				
median (IQR)	-0.004 (-0.46, 0.82)	1.24 (0.29, 3.01)				

^a^The significance threshold for difference was set at a P value less than 0.00625 (0.05/8) for multiple comparison correction.

^b^Statistically significant.

## Discussion

MRI and clinical features of TNBC have been studied widely [8,9,13–18] as early non-invasive detection prior to treatment could aid in better prognostic outcomes.

The present study addressed the clinicopathologic and MRI features of TNBC and compared them to those of ER-positive subtype BC. TNBC displayed poorer histologic grade and higher Ki-67 expression (p<0.001) compared to ER-positive subtype BC. This finding was consistent with a previous study by Krizmanich-Conniff et al. [13], which found that a higher pathologic grade of TNBC, and two other studies [14,19] showing higher Ki-67 expression as an independent predictor of TNBC.

Prior studies also found that TNBC is more likely to develop recurrence than other breast cancer subtypes (30,31). In the current study, we observed significantly more recurrence in TNBC than ER-positive subtype BC (p = 0.035) on univariate analysis, but the difference was not maintained in the multivariate analysis (*p* = 0.293)

TNBC is known to manifest as larger lesions than other subtypes [9,20–22], but we saw no significant size difference between TNBC and ER-positive subtype BC in this study.

DWI offers valuable imaging parameters, since it is non-invasive, does not require contrast agents or ionizing radiation, and is quantitative and repeatable. [23] Kang et al. [12] reported that a high-signal rim on DWI within a breast lesion is associated with malignancy. On DWI, we expected rim sign in the periphery of TNBC tumors due to higher cellularity, but we found no significant difference in DWI rim sign between the two breast cancer subtypes. This finding suggests that DWI rim sign may only be a useful parameter in differentiating malignant from benign lesions, but not in differentiating various subtypes of breast cancer.

In general, malignant breast lesions display a lower ADC value because of high cellular density that results in restriction of diffusion of water molecules. [24,25] However, prior studies [8,22] have found that TNBC has a higher mean ADC value than other breast cancer subtypes due to TNBC's necrotic components. Our results confirmed findings of higher mean ADC value (P<0.001) in TNBC in a univariate analysis. Furthermore, we mapped ADC histograms and found several ADC parameters (mode, 25th and 50th percentiles, and kurtosis; all P<0.001) to be higher than those of the ER-positive subtype in univariate analysis. However, only ADC kurtosis, which indicates a measure of the histogram peak, was found to be significantly higher in TNBC than ER-positive subtype BC (p<0.001) in multivariate analysis. Higher kurtosis reflects a sharper peak and wider tails of the distribution of ADC values. [26] Various studies have found that kurtosis of ADC histograms has implications in evaluating cell differentiation and heterogeneity of measured lesions. [27–31] With respect to cell heterogeneity, Guan et al. [27] found that the normal cervix displays significantly lower ADC kurtosis than cervical cancer because of the homogeneity of normal tissues. Shindo et. al [30] reported that pancreatic adenocarcinoma has a higher kurtosis than neuroendocrine tumors, demonstrating that adenocarcinoma is more heterogeneous, since neuroendocrine tumors are known to be more homogeneous. We assume from such findings that the higher ADC kurtosis of TNBC could imply that it has heterogeneous internal components. As for cell differentiation, higher kurtosis is associated with higher stage bladder cancer [28], poorly differentiated gastric carcinomas [29], and higher grade pancreatic neuroendocrine tumors [31] compared to their well-differentiated counterparts. Therefore, higher ADC kurtosis of TNBC is consistent with poorer cell differentiation, reflecting a more aggressive disease course. A possible explanation for this phenomenon is that normal cell structures are more well-maintained, while those of poorly-differentiated lesions have disordered tumor cells that cause disproportionate distribution in the histogram with a concentration on low ADC values.

On top of the inherent limitations of the retrospective design, our study carries a few additional limitations. First, small tumors less than 1cm and non-mass enhancement lesions were excluded due to limited quality of reconstructed image from DWI and difficulty in manual selection. In addition, the ADC values of non-mass enhancement lesions were reported to be higher than those of mass lesions even in the invasive ductal carcinoma[11] because normal parenchymal tissue was easily included. Considering the incidence of small lesions and non-mass enhancement lesions, this could limit the clinical applicability of our resuts. Our study data had relatively more incidence of TNBC cases than the general population because exclusion of non-mass enhancement lesions might have caused more exclusion of ER-positive subtype than TNBC subtype that often displays mass lesions. Furthermore, our institution is a referral center that draws younger patients and those with poorer breast cancer prognosis from primary clinics, which in turn leads to more concentration of TNBC patients. These could have added selection bias in our data. Finally, the follow-up period for detection of recurrence was quite short (mean, 34.73±11.98 months; range, 5–55 months), which may explain the relatively small number of recurrent events.

In conclusion, TNBC displayed poorer clinicopathologic outcomes than ER-positive BC. Whole-lesion ADC analysis revealed ADC kurtosis to be higher in TNBC than the ER-positive subtype, indicating that it may be a useful index for differentiating TNBC from other breast cancer subtypes.

## Supporting information

S1 Dataset This is the basic dataset of this study including pathologic and radiologic information.
(XLSX)
